# Evaluation of pre-, per- and postoperative data for comparison of short and midterm results of high-risk coronary arterial bypass surgery in patients with severe left ventricular dysfunction

**DOI:** 10.1186/1749-8090-8-S1-P100

**Published:** 2013-09-11

**Authors:** M Akyuz, B Lafci, U Yetkin, M Bademci, B Ozpak, I Akyildiz, B Ozcem, A Gurbuz

**Affiliations:** 1 Department of Cardiovascular Surgery, Izmir Katip Celebi University Ataturk Training and Research Hospital, Izmir, Turkey; 2 Department of Cardiovascular Surgery, Tekirdag State Hospital, Tekirdag, Turkey; 3 Department of Cardiology, Izmir Katip Celebi University Ataturk Training and Research Hospital, Izmir, Turkey

## Background

This study was performed in order to evaluate the short and middle term results of high-risk isolated and elective coronary artery bypass grafting (CABG) surgery in coronary artery disease patients with severe left ventricular dysfunction (preoperative EF≤%30) between February 2010 and April 2012 in our clinic.

## Methods

Preoperative data of the patients were determined in terms of gender distribution, mean age, CCS angina and New York Heart Association (NYHA) functional classification distribution, average EF, distribution of number of arteries with lesion, surgical indication distribution and average euroscore. Accompanying conditions such as chronic obstructive pulmonary disease, diabetes mellitus, hypertension, smoking, obesity, peripheral artery disease, cerebrovascular disease, renal insufficiency, hyperlipidemia and history of PTCA-ICD were questioned in preoperative evaluation. Peroperative data of how many by-passes performed, LIMA usage rates, OPCAB rates, duration of cardiopulmonary by-pass and cross-clamp were evaluated. Early postoperative (first 30 days postoperative) and middle term morbidity and mortality rates were evaluated. İABP usage and inotropic support distribution, mortality rate, major complication rates, average duration of staying in intensive care unit and hospital were monitorized postoperatively. The patients were revisited at 1st, 6th and 12th months after discharge.

## Results

The ejection fraction (EF), left ventricular end-systole diameter (LVESD), left ventricular end-diastole diameter (LVEDD), angina according to CCS classification, and exertional capacity according to (NYHA) classification were evaluated for statistical significance in preoperative and 6th and 12th month postoperatively by transthoracic echocardiographic examination.

## Conclusions

Evaluating our findings, left ventricular functions and clinical findings were improved in patients after high-risk coronary artery by-pass surgery.

